# Utilization of Random Forest and Deep Learning Neural Network for Predicting Factors Affecting Perceived Usability of a COVID-19 Contact Tracing Mobile Application in Thailand “ThaiChana”

**DOI:** 10.3390/ijerph19106111

**Published:** 2022-05-17

**Authors:** Ardvin Kester S. Ong, Thanatorn Chuenyindee, Yogi Tri Prasetyo, Reny Nadlifatin, Satria Fadil Persada, Ma. Janice J. Gumasing, Josephine D. German, Kirstien Paola E. Robas, Michael N. Young, Thaninrat Sittiwatethanasiri

**Affiliations:** 1 School of Industrial Engineering and Engineering Management, Mapúa University, 658 Muralla St., Intramuros, Manila 1002, Philippines; aksong@mapua.edu.ph (A.K.S.O.); thanatorn_chu@rtaf.mi.th (T.C.); mjjgumasing@mapua.edu.ph (M.J.J.G.); jdgerman@mapua.edu.ph (J.D.G.); kperobas@mymail.mapua.edu.ph (K.P.E.R.); mnyoung@mapua.edu.ph (M.N.Y.); 2 School of Graduate Studies, Mapúa University, 658 Muralla St., Intramuros, Manila 1002, Philippines; 3 Department of Industrial Engineering and Aviation Management, Navaminda Kasatriyadhiraj Royal Air Force Academy, Bangkok 10220, Thailand; amarit@rtaf.mi.th; 4 Department of Industrial Engineering and Management, Yuan Ze University, 135 Yuan-Tung Road, Taoyuan 32003, Taiwan; 5 Department of Information Systems, Institute Teknologi Sepuluh Nopember, Kampus ITS Sukolilo, Surabaya 60111, Indonesia; reny@its.ac.id; 6 Entrepreneurship Department, BINUS Business School Undergraduate Program, Bina Nusantara University, Malang 65154, Indonesia; satria.fadil@binus.ac.id

**Keywords:** contact tracing, deep learning neural network, random forest classifier, machine learning algorithm, human behavior

## Abstract

The continuous rise of the COVID-19 Omicron cases despite the vaccination program available has been progressing worldwide. To mitigate the COVID-19 contraction, different contact tracing applications have been utilized such as Thai Chana from Thailand. This study aimed to predict factors affecting the perceived usability of Thai Chana by integrating the Protection Motivation Theory and Technology Acceptance Theory considering the System Usability Scale, utilizing deep learning neural network and random forest classifier. A total of 800 respondents were collected through convenience sampling to measure different factors such as understanding COVID-19, perceived severity, perceived vulnerability, perceived ease of use, perceived usefulness, attitude towards using, intention to use, actual system use, and perceived usability. In total, 97.32% of the deep learning neural network showed that understanding COVID-19 presented the most significant factor affecting perceived usability. In addition, random forest classifier produced a 92% accuracy with a 0.00 standard deviation indicating that understanding COVID-19 and perceived vulnerability led to a very high perceived usability while perceived severity and perceived ease of use also led to a high perceived usability. The findings of this study could be considered by the government to promote the usage of contact tracing applications even in other countries. Finally, deep learning neural network and random forest classifier as machine learning algorithms may be utilized for predicting factors affecting human behavior in technology or system acceptance worldwide.

## 1. Introduction

The COVID-19 pandemic has been present for almost 2 years since March 2020. Despite the vaccination programs evident in different countries, the continuous rise of infected people still prevails. The increasing number of infected people is due to the different variants and mutations that caused the COVID-19 virus to be highly transmittable, mortal, and sometimes undetectable [1], as seen in the Omicron variant. The Omicron variant has become evident and continued to spread to different countries, which affected all individuals.

The prominent increase has caused burnout among people [2] especially healthcare professionals [3,4]. Thus, the need to monitor and trace individuals is necessary to reduce exposure and contain the COVID-19 virus spread. Siddiqui et al. [5] justified that people who are knowledgeable will be practicing preventive measures, however, there is only a weak correlation. Thus, the need to mitigate the COVID-19 virus through monitoring and tracing should be explored.

The COVID-19 tracking and monitoring application/method is available in different countries. In Europe, Kahnbach et al. [6] showed that there is evidence of high functionality, information quality, and esthetics. However, the engagement–orientation quality was relatively weak. In the United Kingdom, Velicia-Martin et al. [7] showed that perceived ease of use would lead to perceived usefulness and attitude. Moreover, in Germany, Behne et al. [8] showed that the application should have an agile set-up and have faster updates towards changes. There are other available contact tracing applications worldwide, however, the need to still consider numerous factors were evident to promote and make the application usable among people in different countries [9]. Despite the availability of kinds of literature regarding tracing applications, there were limited to none regarding “Thai Chana” tracing application from Thailand.

Thai Chana is the main contact tracing application from Thailand [10]. It is a self-reporting online tool for contact tracing among Thais. Thailand was able to consider strategies such as surveillance, laboratory testing, case management and control, risk communication, preparation of healthcare staff, facilities, and medical supplies [11]. Thai Chana helped a lot in the different strategies formulated by Thailand [12]. It has been required among Thais to be utilized when entering a vicinity. It is stated that the government implemented strict compliance to register with the Thai Chana mobile application, applicable to everyone in Thailand, even the foreign visitors. Thai Chana has the capability to gather information such as name, age, addresses, and contact numbers. In addition, it could indicate and transmit information whether someone infected has been in the area. To which, people are guided whether an area is safe, even the need to isolate, and take the test to mitigate the spread of the virus. However, Bangkok, the capital of Thailand is still considered one of the most highly infected cities after China [12]. Thus, the need to explore Thai Chana is important to promote usage and would mitigate the infection rate in the country [13].

Research Questions:

1. Would the integrated Protection Motivation Theory and Technology Acceptance Model holistically measure perceived usability of a health-related application for COVID-19 contact tracing?
2. Could a machine learning algorithm solely measure and predict factors affecting human behavior, specifically measuring perceived technology usability?
3. Are the integrated deep learning neural network and random forest classifier enough to highlight the significant factors affecting perceived usability of a technology?
4. Could the proposed model and methodology be applied and extended to different studies involving human behavior?

To measure the usability of tracing applications such as Thai Chana, frameworks such as Protection Motivation Theory and Technology Acceptance Model could be utilized. Protection Motivation Theory is a fear and coping appraisal theory that is utilized to measure health-related measures [14]. Technology Acceptance Model on the other hand is utilized for measuring the usage of a product or technology [15]. Both studies of Ong et al. [14] and Prasetyo et al. [15] integrated the respective theories to holistically measure an individual’s intention or usage behavior. Protection Motivation Theory solely measures a person’s perception of vulnerability and severity [16]. Van Bavel et al. [17] considered Protection Motivation Theory to measure the minimization of risk and exposure to improve online security behavior. Mousavi et al. [18] considered Protection Motivation Theory for privacy protection behavior on social networking sites. Their results showed that privacy assurance played a significant role in people’s usage of a system.

Consequently, the Technology Acceptance Model was considered by several studies for actual usage of a system. Tomczyk et al. [19] integrated health behavior changes and the Technology Acceptance Model to measure the predicted adoption intentions of the German contact tracing application. Their study considered hierarchical regression modeling and showed that there was only a marginal increase in the predictive value. Moreover, Velicia-Martin et al. [7] considered Technology Acceptance Model regarding the contact tracing application in the United Kingdom. However, their study utilized partial least square–Structural Equation Modeling. According to Fan et al. [20], Structural Equation Modeling alone cannot measure the most significant factor due to the causal relationship among the framework considered. The farther the independent variables from the dependent variables may cause low to no significance. In addition, Woody [21] stated how a mediating effect may be present which hinders the importance and significance level of latent variables due to connections present in a framework. Thus, it would be best to consider utilizing machine learning algorithms such as deep learning neural network and random forest classifier to have high accuracy of prediction among factors affecting human behavior [22].

This study aimed to determine the perceived usability of the COVID-19 contact tracing mobile application in Thailand, Thai Chana. This was achieved through the integration of Technology Acceptance Model and Protection Motivation Theory to measure the perceived usability of Thai Chana as a contact tracing application in Thailand. This was measured using deep learning neural network and random forest classifier to predict factors affecting the perceived usability of Thai Chana. Specifically, factors such as understanding COVID-19, perceived severity, perceived vulnerability, perceived ease of use, perceived usefulness, attitude towards using, intention to use, actual system use, and perceived usability were considered in this study. This is the first study that considered deep learning neural network and random forest classifier for contact tracing applications. The results of this study would be beneficial for contact tracing applications in different countries as a theoretical foundation for new mobile applications for disease control. Lastly, this framework could also be utilized for another application’s usability among different technologies and its overall acceptance worldwide.

With the trend of research focusing on human behavior dealing with integrated multivariate tools and machine learning algorithms [22,23,24,25,26,27,28], this study highlighted how machine learning algorithm alone can accommodate analysis involving technology usability. Little to no studies were found to deal with studies that considered sole machine learning algorithm tools in analyzing human behavior, specifically perceived usability of technology with integrated theories as the framework. In addition, this study is one of the first studies that provided evidence for analyzing factors affecting technology usability using combined random forest classifier and deep learning neural network. The flow of the paper is as follows: (1) Introduction that covers the literature review, gap, and background of the study, (2) related studies and theoretical framework, (3) methodology, (4) results and validation, (5) discussion, and (6) conclusion.

## 2. Related Studies and Theoretical Framework

### 2.1. Machine Learning Algorithm

The machine learning algorithm has been widely utilized due to the availability of bulk data nowadays. It is a tool used for predicting, classifying, and recognizing patterns among different datasets. Several studies [22,23,24] have utilized machine learning algorithm tools such as decision tree with random forest classifier and neural networks. Milani et al. [25] have utilized random forest classifier in classifying factors affecting professional child removal based on parental factors. Their results presented how random forest classifier could be utilized in analyzing factors affecting human behavior. Moreover, Chen et al. [22] considered random forest classifier for predicting risk evaluation of flood disasters in China. The different studies have proved how random forest classifier compared to the normal decision tree provided better classification accuracy [24,25,26]. 

On the other hand, neural networks have been utilized to determine pattern recognition. Neural networks have been developed utilizing an algorithm based on how the neurons transfer information to the brain. It is said to be advantageous since it produces state-of-the-art results based on large datasets [26]. Yariyan et al. [27] utilized Artificial Neural Network focusing on risk assessment in Iran. Oktarina et al. [28] considered neural networks in predicting damages and casualties of people in Indonesia. However, simple neural networks such as artificial neural networks have reduced capabilities to predict higher accuracy due to limited processing of factors. Utilizing a lot of factors would consider more complex calculations. To which, deep learning neural network would be beneficial since it considers more hidden layers for further processing and calculation of output [26]. However, the disadvantage of which is the optimization process to determine the best activation function, optimizer, and the number of nodes since artificial neural networks are classified as a black box.

Compared to the traditional statistical analysis and multivariate tools such as Structural Equation Modeling, machine learning algorithm has been said to have several advantages that suffice the limitations of Structural Equation Modeling. Fan et al. [20] explained how the traditional Structural Equation Modeling has limitations due to the indirect effect the framework has considered. This reduces the level of significance, and may even bring non-significant results due to the connections among dependent and independent variables. In addition, Woody [21] explained how the mediating effects brought by partial and full mediation cause different significant and insignificant results. Therefore, Structural Equation Modeling could not relatively predict the most significant factor affecting human behavior. As suggested by several studies, machine learning algorithms such as random forest classifier and deep learning neural network could be utilized to predict highly significant factors affecting human behavior [23,24,25,26,27,28]. Thus, this study opted to highlight how machine learning algorithms such as deep learning neural network and random forest classifier could be utilized to evaluate human behavior, specifically factors affecting perceived usability of a technology with integrated frameworks of Protection Motivation Theory and Technology Acceptance Theory.

### 2.2. Theoretical Framework

**Figure 1:**
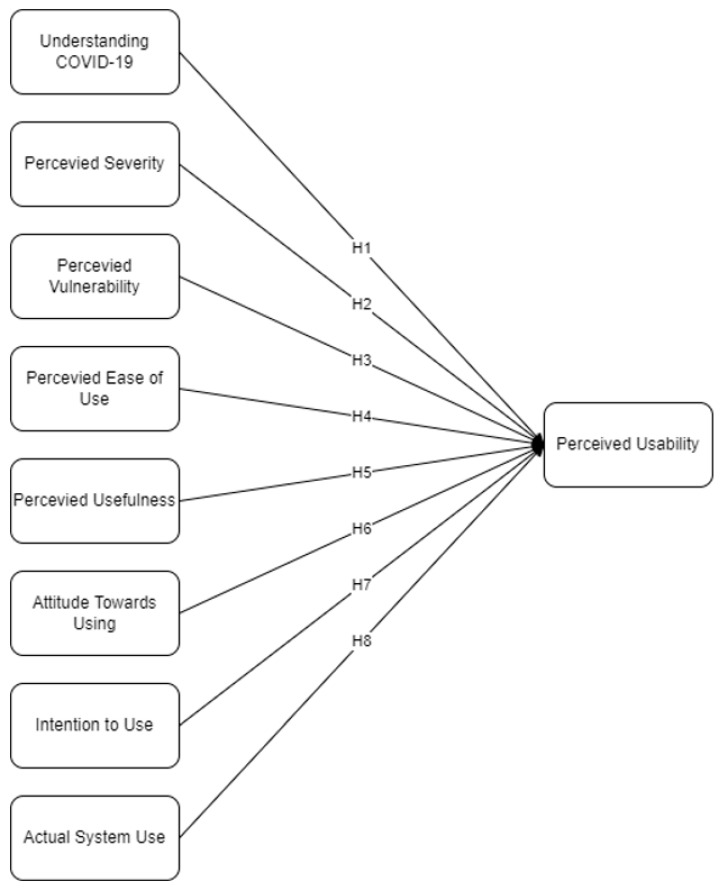
Theoretical Framework.

Presented in Figure 1 is the theoretical framework considered in this study. The integration of Protection Motivation Theory and the Technology Acceptance Model following factors such as understanding COVID-19 (U), perceived severity (PS), perceived vulnerability (PV), perceived ease of use (PEU), perceived usefulness (PU), attitude towards using (A), intention to use (IU), and actual system use (AU) were considered to measure perceived usability (PUS). The basic Technology Acceptance Model factors were considered such as PEU, PU, A, IU, and IU. Protection Motivation Theory is a framework used to measure the threat and coping appraisal influencing the behavioral intention of an individual, dealing with health-related topics [13]. Under Protection Motivation Theory, only those under the threat appraisal factors were considered such as U, PV, and PS. Since “Thai Chana” is utilized for the mitigation of threats brought by the COVID-19 pandemic, the coping appraisal was not included in the framework of this study.

Factors under Protection Motivation Theory when dealing with threats consider different factors such as U, PV, and PS [13,14,15]. Li et al. [29] explained how the perception in choosing and using a mobile application for COVID-19 tracing is caused by people’s risk perception, readiness to use the system, and socialization. With knowledge regarding health-related negative effects, people would often look for ways to mitigate them [14]. Understanding of COVID-19 in this study considers the perception of what the virus is, how it is transmitted, affects, and health protocols. On the other hand, PV is how susceptible the individual is to contract the virus. PS is the perception of how critical the effect of contracting COVID-19 is. Martins et al. [30] explained that knowing the positive effect of mitigation through the use of technology would to an increase in perceived usability. Mingxing et al. [31] also highlighted that when the perception of risks is increased, there is also an increase in the perceived usability of a technology such as contact tracing applications. Thus, it was hypothesized that:

**Hypothesis** **1** **(H1).** *U has the most significant effect on PUS*.

**Hypothesis** **2** **(H2).** *PS has the most significant effect on PUS*.

**Hypothesis** **3** **(H3).** *PV has the most significant effect on PUS*.

PEU is the perception that using a system or technology is free of effort [32]. In addition, PU is defined as the beneficial effect of the technology in the daily activities of an individual [33]. Zheng and Li [34] presented how both PEU and PU are significant latent variables in the acceptance and utility of an application. Mohammadi [32] added that PEU is an influential latent variable that leads to the behavioral intention in using an application. This indicates that if the application is useful and easy to use, then people would have a positive perception of its usability. Especially in health-related applications, Walrave et al. [35] presented how the benefits of an application such as contact tracing would lead to a significant effect on the perceived usability. Therefore, the following were hypothesized:

**Hypothesis** **4** **(H4).** *PEU has the most significant effect on PUS*.

**Hypothesis** **5** **(H5).** *PU has the most significant effect on PUS*.

Attitude, as one of the latent factors in the Technology Acceptance Model, indicates the positive or negative behavior the individual establishes upon using the technology. Parasuraman and Colby [36] explained how the contact tracing application is a new technology, thus the people’s acceptance and perception of usability may be affected due to their attitude in adaptation. Moreover, Li et al. [29] showed how there are negative perceptions in contact tracing adoption due to a pessimistic attitude in using a system. However, the study of Ong et al. [37] explained that when health is at stake, people would consider utilizing technologies, which will lead to a positive perception of how highly usable a technology is. Thus, it was hypothesized that:

**Hypothesis** **6** **(H6).** *A has the most significant effect on PUS*.

IU in this study pertains to the purpose of using an application, while AU is the definite utility of an application [38]. Dehghani et al. [39] studied enabling technologies, their benefits towards health-related concerns, and the goods the technology may provide. Their study showed how both IU and AU affect the perceived usability among individuals when dealing with health-related applications. Following the study of Pal and Vanijja [33], the actual system has been seen to have a significant effect preceded by usability. In addition, Ong et al. [37] explained how people who have high IU would lead to AU due to their perception of usability and usefulness. Moreover, to measure the usability, this study adapted the questions under the System Usability Scale. System usability scale is said to be the widely utilized tool with standardized questions in assessing perceived usability [40]. To which, the following were hypothesized:

**Hypothesis** **7** **(H7).** *IU has the most significant effect on PUS*.

**Hypothesis** **8** **(H8).** *AU has the most significant effect on PUS*.

## 3. Methodology

### 3.1. Questionnaire

Presented in Table 1 are the constructs considered in this study. Different kinds of literature were considered for the adaptation of the different items utilized. Under Understanding of COVID-19 (U), there were 6 items, perceived vulnerability (PV) and perceived ease of use (PEU) has 5, perceived severity (PS) and perceived usefulness (PU) has 7, attitude towards using (A) and intention to use (IU) has 5, actual system use (AU) has 6, and perceived usability (PUS) has 10. The data were available from answering a 5-point Likert Scale.

Prior to the full distribution of the questionnaire, a pilot test was conducted among 150 responses. The responses collected underwent validation using Chronbach’s alpha test. The result presented a value of 0.836, indicating that the questionnaire is valid for full distribution [52,53]. 

### 3.2. Participants

Through convenience sampling, a total of 800 respondents voluntarily answered the survey for the perceived usability of the “Thai Chana” COVID-19 tracing application. Utilizing an online survey, the questionnaire was distributed through different social media platforms due to the strict COVID-19 lockdown implemented. Before responses were collected, a question regarding their utility of the Thai Chana application was asked. Only those who utilized the Thai Chana mobile application were considered since other respondents (250) answered they utilized the paper documents. Thus, 800 valid data were considered. From the collected data, a total of 37,600 datasets were considered (respondents and their responses) to run the deep learning neural network and random forest classifier. 

### 3.3. Machine Learning Algorithm

**Figure 2:**
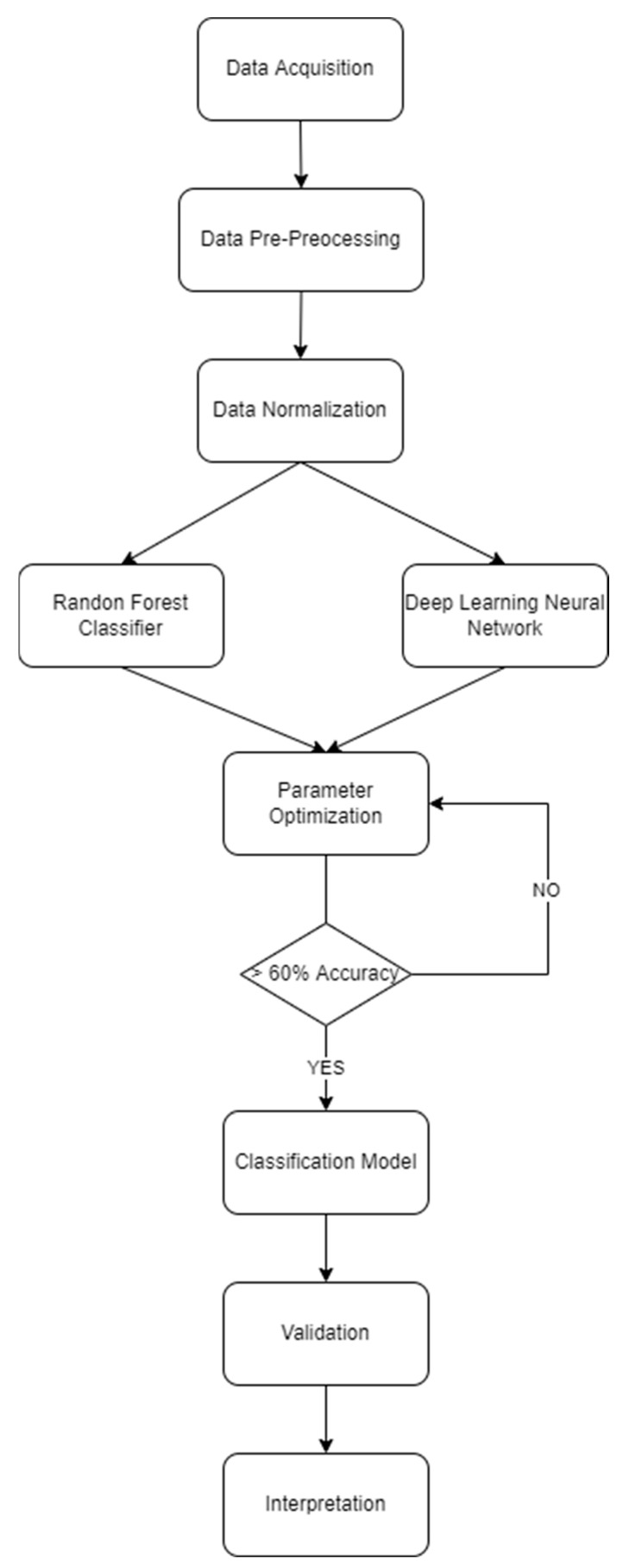
Methodological Flowchart.

Presented in Figure 2 is the methodological flowchart utilized in this study. Data acquisition was carried out through an online survey. Data pre-processing is considered a correlation analysis to determine significant indicators for each latent variable. It was seen that 47 total constructs were collected from the study. Following the study of Kuo and Zulvia [54], those with values less than 0.20 correlation coefficient and *p*-value greater than 0.05 were removed due to insignificance. After which, data aggregation of the 8 latent variables were considered as input parameters for the machine learning algorithm. A min_max scalar normalization technique was utilized for the algorithm [54]. Running the random forest classifier and deep learning neural network, parameter optimization was conducted. Following the study of Chen et al. [22], parameters for criterion, splitter, training and testing ratio, and depth were considered in this study. For the neural network section, the activation functions, optimizer, number of nodes, number of epochs, and number of hidden layers were optimized [55,56,57,58,59]. The following sections provide a detailed explanation for each algorithm.

Following the parameter optimization is the testing for accuracy using cross validation techniques. Following several studies [56,57,58,59], a 60% threshold was set for the accepted accuracy rate of the classification model. After the creation of the final classification model, validation was conducted to test the created models. After which, interpretation was conducted. As support for the utility of machine learning algorithms, no computational complexity was seen. Liu et al. [60] explained how the utilization of complex machine learning algorithms would reduce computation time, complexity, and would have higher accuracy. This study considered Python 3.8 to run all algorithms with SKLEARN and Tensorflow packages. Justus et al. [61] explored the computational complexity of using highly utilized resources and showed that an increase in computation time would be reduced with the current up-to-date technology. From the packages utilized, the main cost of computational complexity would vary from the features used, input data, model complexity, and feature extraction [62,63]. Moreover, the more complex the model is, the more training time [63]. From this study, it was seen that one combination of random forest classifier spent only 0.146 s while deep learning neural network considered 0.480 s. This presents little to no computational complexity with the utilization of the ACER NITRO 5 with core i5 processor, 8 GB RAM, 1 TB Hard disk, Nvidia GeForce GTX 1050 Graphics, running on a Windows 10 system.

### 3.4. Random Forest Classifier

The purpose of using the random forest classifier is to create a classification model that considers different features coming from the constructs which represents the unobserved variables utilized in this study. Following the study of Gao et al. [64], the random forest classifier creates a tree model that considers features that are unified, predicts multi-class dimensions, and presents only significant factors for the classification. The advantage of this algorithm is to present only those significant factors and generate a higher accuracy rate compared to other simple classification models [22]. With the aim to predict and classify factors affecting perceived usability of a new technology among users, random forest classifier may be used to create a classification model for extension and application of predicting influential factors with the integrated theories of Technology Acceptance Model and Protection Motivation Theory. 

For the utilization of random forest classifier, data preprocessing was completed by inspecting missing data. The SPSS 25 indicated no missing data. Following this, data cleaning considering correlation analysis was performed. This study considered a threshold for the correlation value of greater than 0.20 with a *p*-value less than 0.05. From a total of 47 constructs, 34 were considered significant. To which, the data were aggregated to focus on the different factors that influence the usability of Thai Chana application. The factors of U, PV, PS, PEU, PU, A, IU, and AU served as the input nodes for the deep learning neural network. Moreover, PUS served as the output during training.

Data normalization was done and the random forest classifier was utilized considering different parameters such as the criterion (gini and entropy), splitter (best or random), training and testing ratio (60:40, 70:30, 80:20, 90:10), and tree depth (4, 5, 6, 7). Utilizing SKLEARN Packages in Python 4.5, 100 runs of each combination were considered for a total of 6400 optimization runs. It was seen that gini, best, and a 5-tree depth utilizing an 80:20 training ratio presented the highest average accuracy of 92% with 0.00 standard deviation.

### 3.5. Deep Learning Neural Network

Deep learning neural network is known to be ‘the best model’ for predicting factors or recognizing patterns due to its ability to assess and calculate several perceptions [65]. Daube et al. [65] stated how this algorithm resonates with the human-level performance in terms of real-world classification. Luceri et al. [66] added how deep learning neural network could best predict human behavior, social interaction, subjective thoughts, and feeling. Utilizing deep learning neural network in this study would support the result presented from random forest classifier. Since random forest classifier only predicts significant factors, using deep learning neural network could predict and classify the most impactful factors affecting perceived usability of technology in using the Thai Chana COVID-19 contact tracing mobile application. 

Deep learning neural network preprocessing considered data cleaning using correlation analysis, similar to the set conditions with random forest classifier. After data normalization, different activation functions for the hidden layer (sigmoid, tanh, and swish) and output layer (sigmoid and softmax), together with the optimizer (Adam, SDG, RMSProp) was considered. Moreover, the number of nodes was also included for the 80:20 training and testing ratio. To which, a total of 6300 runs were conducted for the feed-forward deep learning neural network process; 10 runs per combination with 150 epochs [56]. This was conducted to determine the best parameters for the deep learning neural network model.

## 4. Results

### 4.1. Participants

The collected data comprised 51.88% male, 45.62% female, and 2.50% others. The majority of which were within 15–24 years old (77.87%), 10.50% were within 25–34 years old, and the rest were 35 years old and above with salaries/allowances less than 15,000 Thailand Bhat (THB) ($454) (41.25%) and 43.12% within THB 15,000–30,000 (USD 454–USD 909.85). Following the suggestion of Ong et al. [14], younger generations are the ones that are most active online, thus justifying that the majority of the respondents are within this age group. In Thailand, most of the younger generation considers using technology easy compared to the older generation that utilizes the document form, since they do not have mobile phones [57]. Lastly, 60.50% were enrolled in insurance and the rest were not. Presented in Table 2 is the summary of the descriptive statistics of the demographics.

In addition, the descriptive statistics of the responses are presented in Table 3. The mean and standard deviation of each indicator is presented. 

### 4.2. Machine Learning Algorithm

**Figure 3:**
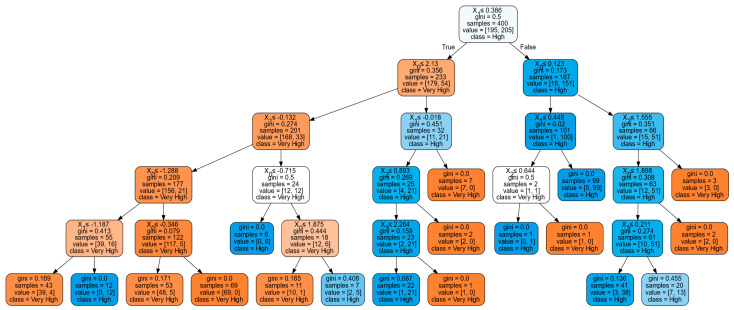
Optimum Random Forest Classifier.

Figure 3 presents the optimum decision tree considering random forest classifier. It could be seen that at 5-tree depth, gini as the criterion, and best as splitter, the random forest classifier produced the highest average accuracy at 80:20 training and testing ratio. With 92% average accuracy from testing and 0.00 standard deviation, this tree was considered for the interpretation of results. The result for random forest classifier was set to very low, low, neutral, high, and very high usability following the 5-point Likert Scale from the survey. To which, these correspond to strongly disagree (1) to strongly agree (5). 

The result indicates that PV (X_1_) will present as the parent node which will lead to perceived usability. The parent will then consider U (X_0_) if true, which will consider both PV and U, leading to very high PUS. If U will not be satisfied, it will consider PS (X_2_) and PEU (X_3_) which will lead to high PUS. Thus, it could be deduced that if people understand COVID-19 and see the vulnerability, Thais would consider Thai Chana very usable. On the other hand, if people know the severity and there is an ease in using the application, then Thais would agree that Thai Chana is usable. Therefore, these factors would lead to the perception of usability of Thai Chana as a COVID-19 contact tracing application. However, the most significant factor could not be indicated since the tree only indicates the path leading to the classification of significant factors. To verify the significance level of the factors, neural network may be applied to show the score of importance among the different significant factors. Abiodun et al. [56] explained how applications of artificial neural network such as deep learning would help in predicting the most significant factor affecting cognitive computing.

Presented in Table 4 is the summarized result for the initial optimization of deep learning neural network. With a 60% threshold for the average accuracy [55,56,57,58,59] of the deep learning neural network, only U, PS, and PV were considered to be significant within the threshold set.

The results obtained from the initial optimization undergo Analysis of Variance (ANOVA) to determine the significant differences among the different factors (U, PS, and PV). The factor causing the significant difference is considered for the final optimization to determine the average accuracy for predicting factors affecting perceived usability of Thai Chana COVID-19 tracing application. U is seen to be the most significant factor with no overfitting.

**Figure 4:**
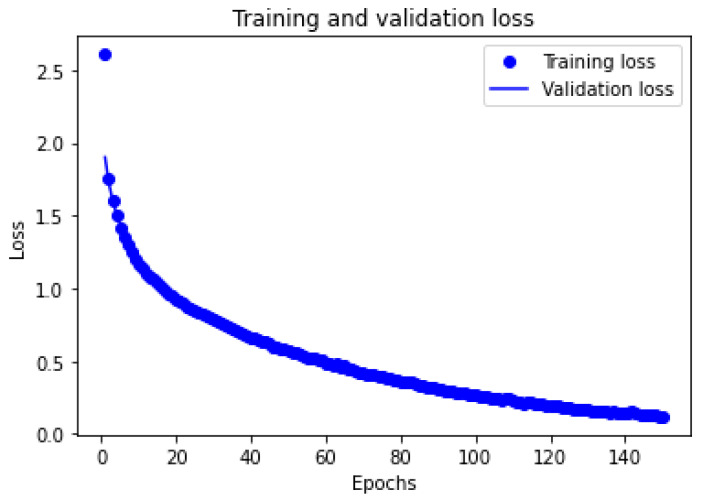
Training and Validation Loss of Deep Learning Neural Network.

The final optimization is conducted with parameters of swish for the hidden layer activation function (30 nodes and 10 nodes) and softmax for the output layer. With adam as the optimizer, the deep learning neural network was run with 200 epochs, 50 times each, for both 70:30 and 80:20 training and testing ratios [59]. The results indicate the highest average accuracy of 97.32% with 1.632 standard deviations for U with an 80:20 training testing ratio. Figure 4 presents the training and validation loss of the final optimization. Based on the result, no overfitting is present [59,64]. Walczak and Cerpa [58] considered this result as relatively high for acceptability with human behavior studies.

**Figure 5:**
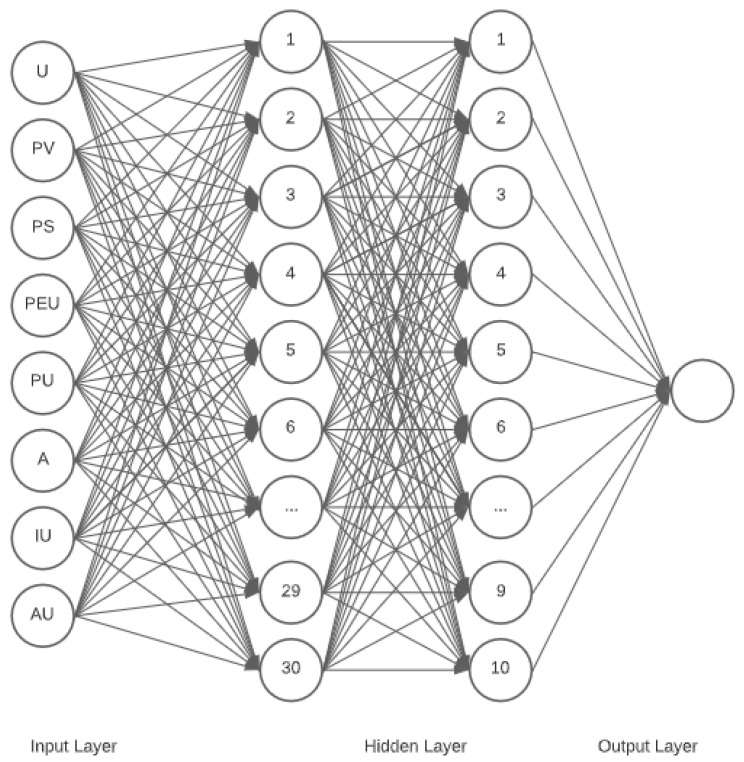
Optimum Deep Learning Neural Network structure for Perceived Usability of Thai Chana COVID-19 Tracing Application.

Figure 5 represents the final deep learning neural network utilized in this study. Based on the figure, the input layer considered 8 nodes (factors) with 30 and 10 nodes for the hidden layers. The activation function of swish and sigmoid with adam as optimizer produced the highest average testing accuracy of 97.32%. It could be deduced that U is the most significant factor affecting the perceived usability of Thai Chana COVID-19 tracing application.

The score of importance is presented in Table 5. Based on the results, U had the highest score of importance (100%), followed by PS (87.5%), PV (77.2%), then PEU (66.0%). Other factors were considered significant but presented a significantly lower score of importance (>60%).

To verify the findings, Pearson’s correlation analysis was conducted, utilizing SPSS V26. Table 6 presents the result from the correlation analysis and presented that U, PS, PV, and PEU were highly correlated with PUS. All of the correlation coefficients presented significant values (*p*-value < 0.05).

## 5. Discussion

This study utilized deep learning neural network and random forest classifier for predicting factors influencing the perceived usability of Thai Chana COVID-19 tracing application. Factors such as understanding COVID-19 (U), perceived severity (PS), perceived vulnerability (PV), perceived ease of use (PEU), perceived usefulness (PU), attitude towards using (A), intention to use (IU), and actual system use (AU) were considered to measure perceived usability (PUS). Based on the result, a 97.32% average accuracy from deep learning neural network was seen and a 92% average accuracy with a 0.00 standard deviation from random forest classifier. Deep learning neural network showed that U had the highest significant effect, followed by PS, PV, and PEU. Consistent with the result from random forest classifier, U and PV led to very high PUS while PS and PEU led to high PUS.

Understanding COVID-19 was seen to be the most significant factor affecting PUS for both deep learning neural network and random forest classifier. The indicators included Thais’ understanding of the COVID-19 virus, its incubation period, symptoms, protocols, when to get the vaccine, and which hospital to go to. The comparison of Asian counties was considered by Wang et al. [67]. Their study showed that Thais have an 89.8% positive belief regarding the knowledge about COVID-19 and that they know its effect when they are in contact. Caldwell et al. [68] explained how the knowledge with the minimum health standard could help in the reduction of COVID-19 transmission. In addition, Ning et al. [69] explained how the success of healthcare professionals regarding interventions would have a positive effect when people know, accept, and understand the disease. Relating to this study, the more people would understand and know about the COVID-19 virus, the more likely they will have the intention to prevent any contraction.

Second, PS was seen to be significant towards PUS among Thais. The indicators included Thais finding of the seriousness of the disease, affects people’s mental health, prolong the outbreak, and that Thailand is more severe than other ASEAN countries. In relation to the study of Fragkaki et al. [70], when people have a higher perception of severity and high government satisfaction, the more likely they will exhibit behavior change. Based on the results, PS was seen to be the second-highest significant factor. Thus, it could be instigated that Thais a have higher perception of severity, leading to the significant factor affecting the PUS of contact tracing application for reduction of exposure. Mant et al. [71] explained that when people have high PS, they would change their behaviors to mitigate COVID-19 transmission. For this study, people will have high PUS when they have high PS of the COVID-19 virus. As a support, this result is also consistent with the findings from Trkman et al. [72].

Third, PV was seen to be a significant factor affecting PUS. The indicators included Thais’ belief in their vulnerability to COVID-19 personally (self, friends, and family), location, and that their country is more vulnerable compared to other ASEAN countries. With that, people have high levels of PUS for the Thai Chana COVID-19 tracing application since they know it will help mitigate the contraction of COVID-19. Boyraz et al. [73] explained that PV positively affects people’s worries, traumatic experiences, and stress. To which, the more vulnerable the perception is, the higher the will to mitigate contraction. This is in line with the results from De Coninck et al. [74] from Belgium. They explained how greater health measures were seen in protecting the population when there is a greater belief in PV. Similar to the results of Ong et al. [37], when people understand the risk, they would highly consider the PV. This would lead to the mitigation in reducing any health-related risks.

Lastly, PEU had a significant effect on PUS among Thai. They believe that Thai Chana as an application can provide clear and understandable information related to COVID-19, successful usage of the application every time, and that the application is easy to use. When utilizing an application, the PEU is usually considered a significant factor [15]. Prasetyo et al. [15] explained how PEU can affect the intention to use a certain application. PEU was also seen to be one of the most significant factors affecting the acceptance and usage of technology [75,76].

Interestingly, PU and A were not considered significant factors affecting PUS. Guillon and Kergall [77] explained how the attitude of a person directly correlates with their belief towards the advantage of quarantine. In addition, Guillon and Kergall [77] explained how trust in the government and health consequences would lead to a high willingness to utilize contact tracing applications. In relation to this study, as long as there is high PS and PV, people will continue to utilize the Thai Chana contact tracing application despite the PU and A. Thus, the advantage of mitigation could be said to revolve around the risk and severity of contracting the virus rather than PU and A. This also supports why IU and AU were not significant. The reason why contact tracing applications are utilized is because of the advantages when it comes to health-related concerns [78,79]. The reason for mitigation upon utilizing the contact tracing application among Thais is for reduction of COVID-19 virus contraction, reduce exposure, and safety in general.

Overall, it could be deduced that when people understand COVID-19, as well as its severity and vulnerability, it would affect their perception of the usability of the Thai Chana COVID-19 tracing application. It could be generalized that when there is risk and health-related concerns, people would understand the benefit of utilizing contact tracing applications. Moreover, as long as there is perceived ease of use, then people would continuously utilize the application. Therefore, this should be considered to promote the utilization of contact tracing applications, not only in Thailand, but may also be applicable to other contact tracing applications worldwide.

### 5.1. Theoretical Implication

The utilization of the machine learning algorithm for human behavior was seen to have different advantages. Machine learning algorithms, such as the artificial neural network, is a type of artificial intelligence that mimics how the body sends a signal to the brain through different neurons that create an output [79]. Deep learning neural network is a type of neural network that has two or more hidden layers that can process the information further and produce higher accuracy [54]. Moreover, Vasilev et al. [80] deep learning neural network has a higher power when it comes to calculation. Ais has been utilized during the COVID-19 pandemic to help in screening, tracking, and predicting future events [80,81]. In addition, Jamshidnezhad et al. [82] utilized a machine learning algorithm for the transmission rate for COVID-19 outbreaks in Iran. Thus, it could be inferred that utilizing machine learning algorithms, such as the deep learning neural network or random forest classifier, may contribute to help mitigating and reducing contraction during the COVID-19 pandemic.

With the utilization of deep learning neural network, it was seen that there was a high accuracy of 97.32% in predicting perceived usability on contract tracing applications. Thus, it could be deduced that the accuracy of prediction is highly reliable. In addition, the 92% accuracy of the srandom forest classifier having consistent results with deep learning neural network further justified the findings. Juarez-Orozco et al. [83] and Chen et al. [22] explained how random forest classifier can be a powerful predictive machine learning algorithm tool for human behavior with higher accuracy since it determines the optimum tree among other decision trees produced. Combining both the result for neural network and random forest classifier would therefore be beneficial in predicting human behavior [22].

### 5.2. Practical Implication

It was seen from the validation that the results presented well-grounded output in using machine learning algorithms to assess and predict factors affecting human behavior, specifically perceived usability of a technology. Academically, the methodology may be applied and extended in related fields of studies, such as assessing behavior and predicting factors applying machine learning algorithms for classification. It could be deduced that integrating multivariate tools with machine learning algorithms or solely using a machine learning algorithm may be utilized to holistically measure and predict human behavior. Recognizing patterns may be evaluated with the consideration of constructs, and measure items to assess unobserved variables. 

Applying the findings of this study, the government may consider implying the severity and vulnerability of COVID-19 in Thailand, comparing it to other countries, and help people understand the COVID-19 virus [84]. This would promote how advantageous Thai Chana as a contact tracing application is. In addition, the usability of Thai Chana was seen to be easy among Thais. This means that there may be only a few changes needed to be made to promote usage. Based on the findings, it could be seen that the highlight on severity and vulnerability may be capitalized on to enhance the perception of usability, increase motivation, and enhance the Thai Chana COVID-19 contact tracing mobile application’s applicability every day. People already understand the implication, side-effects when infected, negative effects, and health complications when infected with the virus. Thus, there is only a need for highlighting the vulnerability and severity in promoting the mobile application. Moreover, the government may enhance motivation by indicating the benefits, both community-wise and health-wise. In addition, the government may also promote the utilization of Thai Chana by campaigning and addressing the public’s concerns. The findings of the study from Munzert et al. [43] showed that people questioned the effectiveness of different contact tracing applications when they are not promoted properly. Therefore, the need to highlight the use, its intention, and application would help people in using the Thai Chana COVID-19 contact tracing mobile application. 

### 5.3. Limitations

This study may have attained a high accuracy rate for both machine learning algorithms, however, there are still limitations. First, this study was conducted only in Thailand and focused on the main contact tracing application, Thai Chana. Other contact tracing applications were not considered. Future researchers may consider other contact tracing applications and compare them to determine the significant factors to generalize the findings. In this way, the contact tracing application may be generalized to enable promotion and usage [82]. Second, this study only considered two machine learning algorithms, deep learning neural network and random forest classifier. Other machine learning algorithm tools may be considered and compared to promote the utility of other algorithms applicable [65]. Classification tools such as support vector machine, and optimization techniques such as particle swarm optimization may be applied. In addition, the location was not considered. The urban and rural areas may have different results depending on their understanding of COVID-19 [85,86,87], perceived severity, and perceived vulnerability towards utilizing the Thai Chana contact tracing application. Thus, clustering may also be applied to enhance the findings of the study, such as KMeans algorithm or Fuzzy CMeans. Lastly, the majority of the age group considered in this study was between 15 and 24 years old only. Due to the COVID-19 lockdown implemented, an online questionnaire was only utilized. As explained by Ong et al. [14], most of the generation utilizing social media platforms are at a younger age. Thus, it is recommended to consider in-person data collection to consider broader and wider age groups. Moreover, findings may be different based on the effect of the virus, perception, and even health-related disease outcomes among the different age groups. Moreover, interviews may be conducted to consider the qualitative measures applicable in measuring perception, knowledge, understanding, and usability errors. A qualitative–quantitative approach may then be utilized to highlight and generalize the perceived usability of Thai Chana as a COVID-19 contact tracing mobile application. This may produce results that may be utilized for workshops, and even create possible techniques that are applicable to other studies.

## 6. Conclusions

The progress in mitigating the COVID-19 contraction has been slow. Due to new variants and the mutation of the COVID-19 virus, the vaccination program was not able to keep up with the progression. To which, countries implemented contact tracing applications to help reduce exposure to the COVID-19 virus [88,89,90,91]. However, there is a lack of study regarding the different applications available. Specifically, the Thai Chana COVID-19 contact tracing application has been underexplored. Thus, this study aimed to predict factors affecting the perceived usability of Thai Chana in Thailand integrating Protection Motivation Theory, Technology Acceptance Model, and System Usability Scale using deep learning neural network and random forest classifier. Specifically, factors such as understanding COVID-19 (U), perceived severity (PS), perceived vulnerability (PV), perceived ease of use (PEU), perceived usefulness (PU), attitude towards using (A), intention to use (IU), actual system use (AU), and perceived usability (PUS) were considered in this study.

With the 800 voluntary Thais participants in the survey, a total of 37,600 datasets were considered. Applying the deep learning neural network, the results produced an average accuracy of 97.32% and 92%, with 0.00 standard deviation for random forest classifier. From the results, U was seen to be the most significant factor, followed by PV, which leads to high PUS. Moreover, PS and PEU were considered significant with high PUS among Thais in using the Thai Chana contact tracing application. When people understand the COVID-19 virus, their perception of severity, and perceived vulnerability would lead to a high perception of contact tracing usability. This means, in order to promote the utilization, the government should instill awareness of the severity and vulnerability among people of the COVID-19 virus. This will also help people to understand how the virus can affect them, leading to the continuous usage of Thai Chana. With that, the government may capitalize on the finding of this study to promote motivation for continuous usage of the mobile application. When people see how the mobile application may help promote positive health-related behaviors and highlight the effect of reduced vulnerability and severity, an increase in motivation for the utility would be applied. As explained by Siddiqui et al. [5], people who are knowledgeable will practice promoting mitigation and intention to reduce the negative effects of COVID-19. In addition, Chuenyindee et al. [13] explained how implementing the utilization of technology does not necessarily promote positive usage. Thus, with the proposed suggestions, people will be highly motivated to use the system.

The consideration of the machine learning algorithm may be highlighted in this study. With high accuracy rates, it was validated that the machine learning algorithms may be utilized for assessing and predicting factors affecting human behavior, not just in assessing technology usability. The disadvantage was the time it took to finish the optimization process for the different parameters to enhance the accuracy of the models considered. In addition, the advantage is the model and parameter setting that was considered, which could be utilized for other studies considering human behavior. In addition, despite several factors considered, the high-quality machine learning algorithms were able to assess the study effectively. Thus, machine learning algorithms could be used to assess perceived usability of technology and other related studies such as natural disasters and even education [90].

The framework and findings of this study may be considered by other countries for their independent contact tracing application. It was seen that when the citizen understood the impact of the virus, how it could affect the health of the people negatively, and how the contact tracing mobile application would help in mitigating the negative side-effects, then people would be more likely to consider and utilize a system to reduce it. It could be deduced that the integrated framework holistically measured health-related technology, its application, and usability among user. Moreover, this may be considered to promote the utilization of contact tracing applications, not only in Thailand [91], but may also be applicable to other contact tracing applications worldwide. Lastly, future researchers may consider the method and framework utilized in this study for the evaluation of applications and systems worldwide.

## Figures and Tables

**Table 1:** Construct and measurement items.

Construct	Items	Measures	Supporting References
Understanding of COVID-19	U1	I do understand the transmission of COVID-19	Prasetyo et al. [41]
	U2	I do understand the incubation period of COVID-19	Li and Lin [42]
	U3	I do understand the general symptom of COVID-19	Munzert et al. [43]
	U4	I do understand the protocol if I have the symptoms that might lead to COVID-19	
	U5	I do understand which hospital can treat COVID-19 patients	
	U6	I do understand when I can get the vaccine for COVID-19 from Thai Government	
Perceived Vulnerability	PV1	I think I am vulnerable to COVID-19	Prasetyo et al. [41]
	PV2	I think my area is very vulnerable to COVID-19	Kowalski and Black [44]
	PV3	I think there is a chance that my family will be infected by COVID-19	
	PV4	I think my friends/colleague is vulnerable to COVID-19	Ong et al. [14]
	PV5	I think Thailand is more vulnerable than ASEAN countries	
Perceived Severity	PS1	I find COVID-19 is a serious disease	Prasetyo et al. [41]
	PS2	I find COVID-19 can lead to sudden death	
	PS3	I find COVID-19 is more severe than other diseases	Kowalski and Black [44]
	PS4	I find COVID-19 can affect my mental health	Ong et al. [37]
	PS5	I think it’s very expensive to pay the medical expenses for COVID-19	Lewis [40]
	PS6	I think the COVID-19 outbreak will continue until the middle of 2021	Walrave et al. [35]
	PS7	I think COVID-19 in Thailand is more severe than ASEAN countries	
Perceived Ease of Use	PEU1	I think Thai Chana can provide information related to COVID-19 that I want	Prasetyo et al. [41]
	PEU2	Information provided by Thai Chana is very clear and understandable	Kurniasih et al. [45]
	PEU3	I can use Thai Chana successfully every time	
	PEU4	I believe the information provided by Thai Chana is correct	Camacho-Rivera et al. [46]
	PEU5	It would be easy for me to become skillful at using Thai Chana	
Perceived Usefulness	PU1	Using Thai Chana would protect me from COVID-19	Prasetyo et al. [43]
	PU2	Using Thai Chana can enhance my health	Kurniasih et al. [45]
	PU3	The COVID-19 spread map can enhance my awareness and preparedness	Camacho-Rivera et al. [46]
	PU4	Safety guidelines in Thai Chana is useful	
	PU5	Announcement in Thai Chana is useful	Gumasing et al. [47]
	PU6	Hotline number in Thai Chana is responsive	
	PU7	Using Thai Chana can safe my community from COVID-19	
Attitude towards using	A1	Thai Chana is beneficial for me	Prasetyo et al. [41]
	A2	Thai Chana makes me feel safe from COVID-19	Kurniasih et al. [45]
	A3	Thai Chana can reduce my stress due to COVID-19	Velicia-Martín et al. [7]
	A4	Thai Chana gives the community a sense of security	
	A5	I feel I have to use Thai Chana for the sake of my health	
Intention to Use	IU1	I will be willing to use Thai Chana in the future	Prasetyo et al. [41]
	IU2	I will continue to use Thai Chana in the future	Kurniasih et al. [45]
	IU3	I will promote Thai Chana to other people in the future	Chuenyindee et al. [48]
	IU4	I will follow the announcement by the government in Thai Chana	
	IU5	I will follow the health protocol in Thai Chana	
Actual System Use	AU1	I intend to install Thai Chana on my device	Prasetyo et al. [41]
	AU2	Most people in my community are using Thai Chana	
	AU3	I feel insecure if I don’t use Thai Chana	Pal and Vanijja [33]
	AU4	I often read announcement in Thai Chana	
	AU5	I follow the safety guidelines provided by Thai Chana	
	AU6	I feel satisfied with Thai Chana	
Perceived Usability	PUS1	I think I would use this system frequently	Prasetyo et al. [41]
	PUS2	I think Thai Chana is unnecessarily complex	Orfanou et. al. [49]
	PUS3	I think Thai Chana is easy to use	German et al. [50]
	PUS4	I think I can operate Thai Chana by myself without the technical support	Pal and Vanijja [33]
	PUS5	I find that various functions in Thai Chana are well integrated	Kuo and Zulvia [51]
	PUS6	I think Thai Chana system is consistent	
	PUS7	I would imagine many people in Thailand will use Thai Chana	
	PUS8	I think it is comfortable using Thai Chana	
	PUS9	I feel confident using Thai Chana	
	PUS10	I do not need to learn many things before using Thai Chana	

**Table 2:** Demographic Profile of Respondents (n = 800).

Characteristics	Category	N	%
Gender	Male	365	45.62
	Female	415	51.88
	Other	20	2.50
Age	15–24	623	77.87
	25–34	84	10.50
	35–44	34	4.250
	45–54	31	3.870
	55–64	27	3.380
	More than 64	1	0.130
Monthly Salary/Allowance	THB < 15,000	330	41.25
	THB 15,000–30,000	345	43.12
	THB 30,000–45,000	65	8.130
	THB 45,000–60,000	30	3.750
	THB 60,000–75,000	12	1.500
	THB > 75,000	18	2.250
Enrolled in a health insurance?	Yes	484	60.50
	No	316	39.50

**Table 3:** Descriptive Statistics of the Indicators.

Construct	Items	Mean	Standard Deviation
Understanding of COVID-19	U1	4.4213	0.70493
	U2	4.2950	0.81934
	U3	4.4150	0.70774
	U4	4.4875	0.68588
	U5	4.0800	0.97969
	U6	3.6688	1.22919
Perceived Vulnerability	PV1	3.1050	1.38162
	PV2	3.3688	1.26284
	PV3	2.9500	1.44486
	PV4	3.2600	1.34058
	PV5	3.7988	1.10670
Perceived Severity	PS1	4.3825	0.84385
	PS2	4.0563	1.03413
	PS3	4.1263	0.93215
	PS4	4.2138	0.94957
	PS5	4.3475	0.88046
	PS6	4.4350	0.76910
	PS7	3.8688	1.09568
Perceived Ease of Use	PEU1	3.8750	1.18475
	PEU2	3.9000	1.08350
	PEU3	3.8750	1.18897
	PEU4	3.9050	1.07467
	PEU5	3.9788	1.09421
Perceived Usefulness	PU1	3.7150	1.28680
	PU2	3.6850	1.26798
	PU3	3.8913	1.13403
	PU4	3.9050	1.13140
	PU5	3.9100	1.16559
	PU6	3.8013	1.17621
	PU7	3.7950	1.22161
Attitude Towards Using	A1	3.9125	1.19546
	A2	3.7375	1.22289
	A3	3.6963	1.23628
	A4	3.8063	1.17065
	A5	3.8100	1.17713
Intention to Use	IU1	3.9525	1.14319
	IU2	3.8600	1.15089
	IU3	3.8250	1.16450
	IU4	3.7688	1.24286
	IU5	3.8500	1.18396
Actual System Use	AU1	3.8000	1.29019
	AU2	3.7025	1.28883
	AU3	3.6650	1.30572
	AU4	3.5500	1.35824
	AU5	3.6850	1.25708
	AU6	3.7225	1.21237
Perceived Usability	PUS1	3.8050	1.20781
	PUS2	3.6000	1.19866
	PUS3	3.8975	1.05872
	PUS4	3.8625	1.07006
	PUS5	3.8163	1.08234
	PUS6	3.8413	1.11696
	PUS7	3.7475	1.14363
	PUS8	3.8600	1.08599
	PUS9	3.8313	1.17239
	PUS10	3.8900	1.04368

**Table 4:** Summary of Initial Deep Learning Neural Network.

Latent	Nodes	Activation (H–Layer)	Activation (O–Layer)	Optimizer	Average Training	StDev	Average Testing	StDev
U	30	swish	sigmoid	adam	32.29	2.063	91.63	3.662
PS	40	swish	sigmoid	adam	24.70	1.663	86.32	2.843
PV	30	swish	sigmoid	adam	13.10	5.032	83.62	4.633
PEU	50	swish	softmax	SGD	33.85	1.630	68.75	5.563
PU	50	swish	softmax	adam	31.11	2.368	36.25	4.478
A	40	swish	softmax	adam	24.21	3.654	48.75	5.001
IU	40	swish	softmax	RMSProp	27.66	1.635	40.23	2.658
AU	30	tanh	softmax	SGD	25.72	2.156	42.24	3.665

**Table 5:** Score of Importance.

Latent	Importance	Score (%)
U	0.213	100
PS	0.186	87.5
PV	0.164	77.2
PEU	0.140	66.0
PU	0.116	54.5
A	0.055	25.7
IU	0.059	27.7
AU	0.067	31.4

**Table 6:** Pearson’s R Correlation.

Latent	U	PV	PS	PEU	PU	A	IU	AU
**PV**	0.392							
**PS**	0.370	0.414						
**PEU**	0.398	0.267	0.282					
**PU**	0.348	0.243	0.315	0.866				
**A**	0.392	0.223	0.274	0.809	0.890			
**IU**	0.207	0.181	0.223	0.780	0.828	0.881		
**AU**	0.144	0.246	0.273	0.787	0.831	0.873	0.898	
**PUS**	0.779	0.715	0.739	0.716	0.715	0.244	0.217	0.308

## Data Availability

The data presented in this study are available on request from the corresponding author.
